# Pedestrian tracking method based on S-YOFEO framework in complex scene

**DOI:** 10.1371/journal.pone.0322919

**Published:** 2025-06-04

**Authors:** Wenshun Sheng, Jiahui Shen, Qi Chen, Qiming Huang

**Affiliations:** Pujiang Institute, Nanjing Tech University, Nanjing, Jiangsu, China; Purdue University, UNITED STATES OF AMERICA

## Abstract

A real-time stable multi-target tracking method based on the enhanced You Only Look Once-v8 (YOLOv8) and the optimized Simple Online and Realtime Tracking with a Deep association metric (DeepSORT) for real-time stable multi-target tracking (S-YOFEO) is proposed to address the issue of target ID transformation and loss caused by the increase of practical background complexity. The complexity of the real-world context poses a great challenge to multi-target tracking systems. Changes due to weather or lighting conditions, as well as the presence of numerous visually similar objects, can lead to target ID switching and tracking loss, thus affecting the system’s reliability. In addition, the unpredictability of pedestrian movement increases the difficulty of maintaining consistent and accurate tracking. For the purpose of further enhancing the processing capability of small-scale features, a small target detection head is first introduced to the detection layer of YOLOv8 in this paper with the aim of collecting more detailed information by increasing the detection resolution of YOLOv8 to ensure precise and fast detection. Secondly, the Omni-Scale Network (OSNet) feature extraction network is implemented to enable accurate and efficient fusion of the extracted complex and comparable feature information, taking into account the restricted computational power of DeepSORT’s original feature extraction network. Again, addressing the limitations of traditional Kalman filtering in nonlinear motion trajectory prediction, a novel adaptive forgetting Kalman filter algorithm (FSA) is devised to enhance the precision of model prediction and the effectiveness of parameter updates to adjust to the uncertain movement speed and trajectory of pedestrians in real scenarios. Following that, an accurate and stable association matching process is obtained by substituting Efficient-Intersection over Union (EIOU) for Complete-Intersection over Union (CIOU) in DeepSORT to boost the convergence speed and matching effect during association matching. Last but not least, One-Shot Aggregation (OSA) is presented as the trajectory feature extractor to deal with the various noise interferences in complex scenes. OSA is highly sensitive to information of different scales, and its one-time aggregation property substantially decreases the computational overhead of the model. According to the trial results, S-YOFEO has made some developments as its precision can reach 78.2% and its speed can reach 56.0 frames per second (FPS), which fully meets the demand for efficient and accurate tracking in actual complex traffic environments. Through this significant increase in performance, S-YOFEO can contribute to the development of more reliable and efficient tracking systems, which will have a profound impact on a wide range of industries and promote intelligent transformation and upgrading.

## Introduction

In the fields of computer vision and artificial intelligence, multi-target pedestrian detection and tracking have emerged as a critical research area with significant practical implications. This technology aims to detect and track multiple pedestrians in real-time within a sequence of images or video frames, enabling a wide range of applications across various domains. The rapid pace of urbanization and technological advancement has further amplified the importance of this research area, leading to its integration into several key fields. In autonomous driving, for instance, multi-target pedestrian detection and tracking systems are essential for enabling vehicles to accurately perceive and analyze other road users, including pedestrians, cyclists, and vehicles, in their immediate surroundings. This capability is crucial for ensuring the safety and reliability of autonomous vehicles in complex urban environments. Similarly, in video surveillance, the ability to detect and track pedestrians in real-time has significantly raised the bar for video analytics, enabling more intelligent and proactive monitoring of public spaces, enhancing security, and facilitating crowd management. Furthermore, in robot navigation, these systems play a vital role in helping robots accurately identify and understand their surroundings, enabling them to navigate safely and efficiently in dynamic environments.

The evolution of target detection techniques has been marked by a significant transition from manual feature extraction to automatic feature learning, driven by the ongoing development of deep learning algorithms. In single-stage detection methods, an image is divided into regions, and each region’s target categories and bounding boxes are predicted simultaneously [1]. This approach has gained popularity due to its simplicity and efficiency, particularly in real-time applications. On the other hand, target tracking techniques have primarily evolved into two categories: deep learning-based methods and correlation filtering-based methods. Both approaches have made substantial progress in addressing key challenges such as target occlusion, abrupt changes in target appearance, and complex backgrounds. However, as traffic environments and urban scenarios become increasingly complex, the demands placed on multi-target pedestrian detection and tracking systems continue to grow. These systems must now be capable of handling a wide range of challenges, including frequent occlusions, dynamic changes in pedestrian density, and difficulties in distinguishing pedestrians from complex backgrounds. These challenges necessitate the development of more robust and efficient algorithms that can operate reliably in real-world conditions.

To address these challenges, this paper presents the S-YOFEO framework, a novel approach designed to enhance the performance of multi-target pedestrian detection and tracking models. The framework leverages the YOLOv8 [2] algorithm as its detector, which is known for its superior performance in terms of computational efficiency and detection accuracy. YOLOv8 is further enhanced by the addition of a small target detection head, enabling the model to capture richer feature information and improve its ability to detect pedestrians in crowded and complex scenes. For tracking, the framework employs an enhanced version of the DeepSORT [3] algorithm, referred to as Optimized Feature Extraction and Occlusion Handling (OFEO). This improved tracker incorporates several innovations, including a new adaptive oblivious Kalman filter algorithm to better model pedestrian trajectories, an OSA architecture to enhance appearance feature extraction, and an EIOU association matching metric to improve the accuracy of target matching. These enhancements collectively contribute to significant improvements in tracking efficiency and accuracy, particularly in challenging scenarios.

The remainder of the paper is structured as follows: the Related work section provides an overview of the relevant literature; the Model design and implementation section details the model’s development and application; and the Experimental results and analysis section presents a comparative analysis of S-YOFEO’s target recognition and tracking efficacy versus other models. An overview and outlook of this study are given in the Conclusion section.

These are the primary contributions of this work:

- A YOLOv8 algorithm with the inclusion of a tiny target detection head is suggested in order to increase the model’s detection accuracy in packed scenarios.
- This study offers a new adaptive oblivious Kalman filter algorithm, the FSA Kalman filter [4], to adapt to the real trajectory of pedestrians.
- This work uses the OSA [5] architecture to improve DeepSORT appearance feature extraction by lessening the effect of different noises produced in complicated surroundings on target tracking.
- This work uses the EIOU [6] association matching metric to measure the match between the detection frame and the prediction frame to increase the accuracy of target matching.

Together, these contributions form the foundation of the S-YOFEO framework, which represents a significant step forward in the field of multi-target pedestrian detection and tracking. By addressing key challenges and introducing innovative solutions, this work aims to pave the way for more reliable and efficient systems in applications such as autonomous driving, video surveillance, and robot navigation.

## Related work

The application of deep learning for multi-target detection and tracking has gained popularity and acceptance in the current research field due to its rapid progress.

A pure transformer architecture called TransFlow has been presented in [7] for optical flow estimation. TransFlow achieves state-of-the-art results on the Sintel, and KITTI-15, as well as several downstream tasks, including video object detection, interpolation, and stabilization.

Unlike TransFlow, the paper [8] introduces Prototypical Transformer (ProtoFormer), a general and unified framework that approaches various motion tasks from a prototype perspective. ProtoFormer achieves competitive performance on popular motion tasks such as optical flow and scene depth and shows broad applicability in downstream tasks, including object tracking and video stabilization.

In [9], Lou H, *et al*. proposed a small-size object detection algorithm for special scenarios in response to the fatigue and cognitive limitations of traditional camera sensors that rely on human eye observation. The advantage of this algorithm is that it not only has higher accuracy in detecting small-sized objects but also ensures that the detection accuracy of objects of various sizes is not lower than that of existing algorithms.

The paper [10] proposes YOLO, which saves a significant amount of computing expenses by fully discarding the two-stage region suggestion and regression detection technique. 45 FPS [11] is the top speed. In contrast to the two-stage detector, the accuracy is only 63.4%, indicating a drop in detection accuracy.

UAV-YOLOv8, an optimized model for the problem of object detection, was proposed by Wang G *et al*. in [12] in Unmanned Aerial Vehicle (UAV) images. Compared with the baseline model, the UAV-YOLOv8 model has fewer parameters, improves the average detection accuracy by 7.7%, effectively improves the detection of small objects, and achieves a better balance between detection performance and resource consumption.

The paper [13] addresses the field of Multi-Objective Tracking (MOT) and proposes a tracker named StrongSORT with significant improvements from multiple perspectives, such as object detection, feature embedding, and trajectory association. By combining StrongSORT with AFLink and GSI, the final tracker (StrongSORT++) achieves state-of-the-art results on multiple public benchmarks, i.e., MOT17, MOT20, DanceTrack, and KITTI.

This technique enhances selectivity and localization prediction framework. According to experimental results, the system is ideal for online real-time pedestrian tracking and can recognize pedestrians even in situations involving rapid quick movement, occlusion, and appearance deformation.

In conclusion, there are certain benefits to combining deep learning and multi-target tracking for pedestrian monitoring. Nevertheless, due to the intricate traffic scene, the model finds it challenging to strike a balance between multi-target tracking’s efficiency and accuracy. This study presents a pedestrian tracking system based on S-YOFEO, which aims to balance the efficiency against the accuracy trade-off.

## Model design and implementation

This paper presents a novel solution to the issues of low accuracy and efficiency during target detection and tracking due to occlusion and too-small targets caused by complex scenes: a multi-target pedestrian tracking model (S-YOFEO) based on the optimized DeepSORT and improved YOLOv8.

### Target detection branch

A single-stage target detection technique called YOLOv8 has been invented based on YOLOv5 [14]. To further increase the network’s sensitivity and accuracy, YOLOv8 encompasses state-of-the-art (SOTA) [15] technology, innovative optimization of the backbone network, detection head, and loss function.

**Fig 1:**
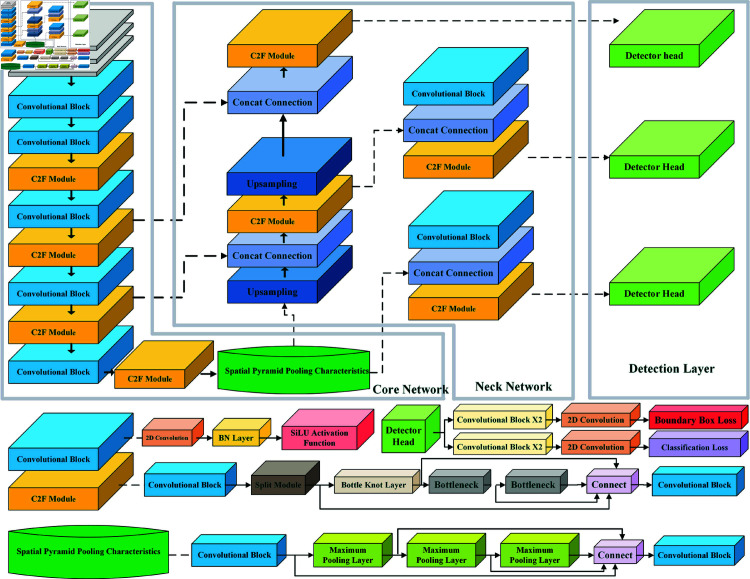
YOLOv8 overall framework.

Fig 1 depicts the general network architecture of YOLOv8, where the decoupling head [16] is chosen as the detection head. The decoupling head can extract all the target location and classification information, learn them separately through the detection network and classification network, and then fuse the information. This is considering the general coupling head recognizes it difficult to perform complex information extraction on the different focuses of target location and classification information. With the implementation of the straightforward branch learning concept, the target object may be completely and effectively acknowledged by extracting its texture content and edge information, successfully reducing the computational cost of the network and preventing overfitting. In addition, it optimizes the performance of the model in terms of generalization and resilience.

**Fig 2:**
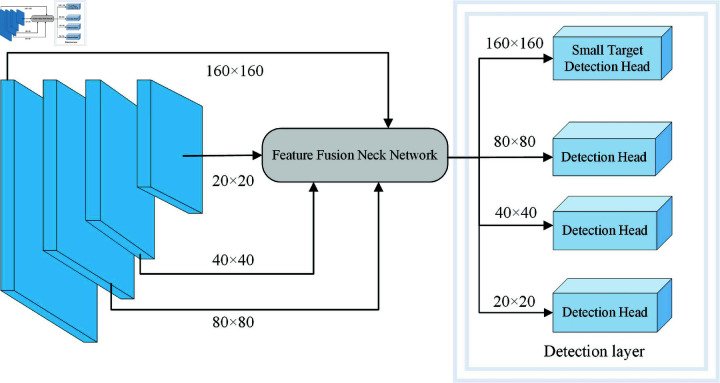
Improved detection layer.

YOLOv8’s recognition of small targets in densely populated pedestrian scenes frequently leads to several issues, including misidentification and omission. This study expands the three previous detection layers to four layers, as indicated in Fig 2, by adding a 160×160 size small target detection head in the detection layer. To better identify the target from the background, the compact target detection head has a greater resolution and has the ability to capture more detail in the image.

### Trace detection branch

**Fig 3:**
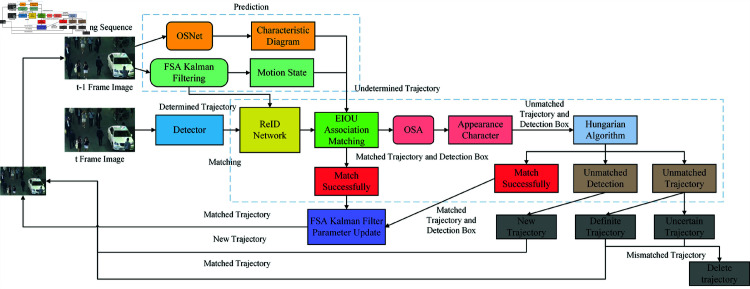
Algorithm flowchart of OFEO.

Based on Simple Online and Realtime Tracking (SORT) [17], DeepSORT is an advanced visual target tracking system that can extract motion and appearance information from the tracked target [18]. However, the performance demonstrated by DeepSORT in such an environment can no longer fulfill the real requirements due to the growing complexity of the tracking situations and the increased gradient nature of state noise during motion and prediction. Because of this, the updated DeepSORT algorithm is referred to as the OFEO tracking algorithm in this research, which also optimizes its prediction and association matching components. Fig 3 displays the algorithmic flowchart for OFEO.

The three primary components of the OFEO tracking algorithm are association matching, feature extraction, and target detection. First, the OSNet [19] network is executed to extract the feature information from the input video sequence of *t*–1 frame. The target trajectory is then directly derived if the matching is successful, and the OSA is applied to the unsuccessful one to complete the extraction of global features and deep information of the image. Afterward, it is matched with the FSA Kalman filter proposed in this paper, and the matching result is associated with the trajectory sequence of *t* frame after the target detection by EIOU, the competing algorithmic problems of accurate tracking such as occlusion and target number transformation due to scene changes are reduced and the tracking accuracy is improved. Lastly, the OSA is paired with the Hungarian algorithm [20] to produce the target trajectory.

#### OSNet feature extraction network.

**Fig 4:**
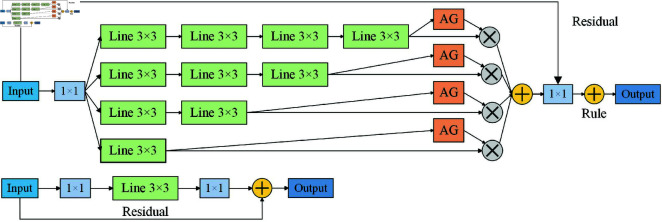
OSNet network structure.

Due to CNN’s low processing power, DeepSORT’s feature extraction network uses a basic convolutional neural network (CNN) [21], which is unable to adapt to highly complex and demanding real-world settings. Consequently, OSNet which facilitates full-scale feature learning is used as the feature extraction network in this work. In addition to its ability to extract target feature information in an omnidirectional and multi-angle fashion, OSNet, as demonstrated in Fig 4, features a lightweight network topology that lowers the computational overhead of the model while preventing the occurrence of network overfitting.

The OSNet architecture, as illustrated in Fig 4, is composed of several residual blocks with convolutional feature streams that capture features at different scales. This mechanism of multiscale feature learning helps the network to understand the input data more comprehensively and extract more discriminative features, which improves the model’s ability to handle complex scenes. To achieve dynamic scale fusion, a structure known as a unified aggregation gate (AG) [22] is used to dynamically assign weights to features at different scales based on the input image. This dynamic scale fusion approach enables OSNet to adapt more flexibly to different input data and extract more representative features, which helps the network to unfold global feature extraction. Additionally, OSNet, due to its lightweight network topology, has lower computational complexity and usually occupies less computational resources and memory space, which enables OSNet to run on resource-constrained devices and is more efficient and convenient in terms of device deployment, which helps to apply OSNet to a wider range of scenarios.

#### FSA Kalman filter algorithm.

The prediction and update phases make up the two primary components of the Kalman filtering technique, which is a constant velocity linear state optimal estimation algorithm [23]. To obtain an a priori estimate of the present instant, prediction involves estimating the state at that moment based on the a posteriori estimate of the preceding moment. On the other hand, updating may be further divided into two categories: measurement updating and time updating. Eq 1 represents the time updating equation for the Kalman filtering technique and Eq 2 represents the measurement updating equation.

$$\left\{ \begin{array}{c} {\hat{x}}_{\overset{―}{t}}=A{\hat{x}}_{t-1}+Bu_{t-1}\\ P_{\overset{―}{t}}=AP_{t-1}A^{T}+Q \end{array}\right.$$ (1)

Where ${\hat{x}}_{\overset{―}{t}}$ denotes the prior state estimate of the *t* moment, ${\hat{x}}_{t-1}$ denotes the posterior state estimate of the *t*–1 moment, *A* denotes the state transition matrix, *B* denotes the matrix that converts the input to the state, *u*_*t*−1_ denotes the input of the *t*–1 moment, $P_{\overset{―}{t}}$ denotes the prior estimation covariance of the *t* moment, *P*_*t*−1_ denotes the posterior estimation covariance of the *t*–1 moment, *Q* denotes the process excitation noise covariance.

$$\left\{ \begin{array}{l} K_{t}=\frac{P_{t}-H^{T}}{HP_{t}-H^{T}+R}\\ {\hat{x}}_{t}={\hat{x}}_{\bar{t}}+K_{t}(z_{t}-H{\hat{x}}_{\bar{t}})\\ P_{t}=(I-K_{t}H)P_{\bar{t}} \end{array}\right.$$ (2)

Where *K*_*t*_ denotes the measurement noise covariance, *H* denotes the observation matrix, *R* denotes the Kalman gain matrix, and *z*_*t*_ denotes the observation value.

Nevertheless, in practical situations, pedestrians’ movement speeds and trajectories are unpredictable, therefore the linear Kalman filter is inconsistent with reality when it comes to target prediction and parameter update. This study introduces a new adaptive oblivious Kalman filter algorithm, the FSA Kalman filter, whose specific steps are illustrated in Algorithm 1. The algorithm is intended to adjust to the movement speed and trajectory state of real pedestrians.

**Algorithm 1. Adaptive forgetting Kalman filter algorithm FSA.**

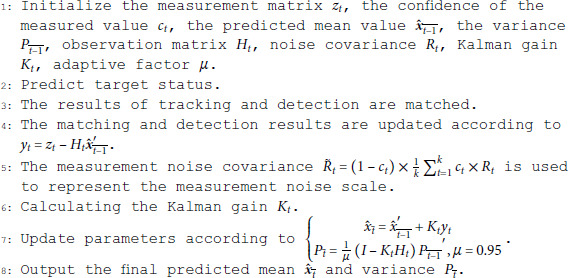



#### EIOU association matching.

The target matching between the predicted and detected locations by the Kalman filtering technique is carried out by the DeepSORT algorithm using the cost matrix and Hungarian algorithm. In an effort to attain improved tracking, DeepSORT employs IOU [24] for correlation matching to ascertain the degree of correspondence between the actual and anticipated detection frames. Equation reveals the IOU Eq 3.

$$IOU=\frac{\left|A\cap B\right|}{\left|A\cup B\right|}$$ (3)

Where the projected target bounding box is represented by *A* and the real target bounding box by *B*.

IOU, however, has certain restrictions in certain situations. For instance, when there is no overlap between the two bounding boxes, the IOU is 0, which also results in the gradient becoming 0, making it impossible to carry out secondary optimization on the data [25]. This study proposes to replace IOU for association matching with EIOU, which performs better for crowded scenes with high object shape similarity. Eq 4 indicates the formula of EIOU. To overcome this challenge, the paper examines four typical loss functions.

$$EIOU=1-IOU+\frac{\rho^{2}\left(b,b^{gt}\right)}{{\left(w_{c}\right)}^{2}+{\left(h_{c}\right)}^{2}}+\frac{\rho^{2}\left(w,w^{gt}\right)}{{\left(w_{c}\right)}^{2}}+\frac{\rho^{2}\left(h,h^{gt}\right)}{{\left(h_{c}\right)}^{2}}$$ (4)

Where ρ denotes the computation of the Euclidean distance between the centers of the predicted frame and the real frame, and *w*_*c*_ and *h*_*c*_ denote the width and height of the smallest outer rectangle that can contain both the predicted frame and the real frame, respectively.

**Fig 5:**
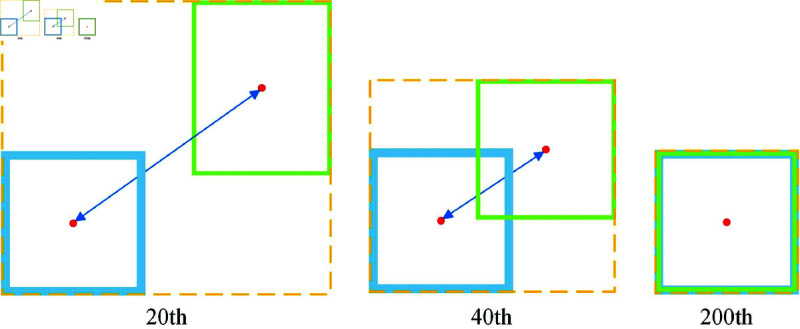
The matching process of EIOU.

The matching effect of EIOU when IOU is 0 is shown in Fig 5.

The model is able to produce better localization results and faster convergence owing to EIOU’s ability to minimize the width and height difference between the predicted and real frames, as shown in Fig 5. This feature significantly increases the efficiency and accuracy of the OFEO algorithm’s association matching.

#### OSA trajectory feature extractor.

**Fig 6:**
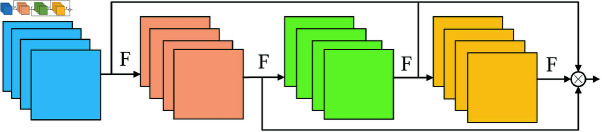
Network structure of OSA.

This research utilizes OSA for trajectory feature extraction to account for different types of noise interferences in complicated situations. By combining feature information from differential-rich sensory fields, OSA strengthens the model’s sensitivity to focus on diverse scales, which may dramatically enhance the network’s extraction of deep semantic information. The network structure is shown in Fig 6.

All features are aggregated only once in the final feature mapping after OSA accumulates the rich feature information [26]. This optimizes the network’s computational and energy efficiency by somewhat diminishing the processing and storage requirements for repetitive features. This innovative design approach effectively minimizes redundant feature processing operations and optimizes storage resource utilization. By eliminating unnecessary repetitive computations and reducing data redundancy, the system achieves substantial improvements in computational efficiency and energy utilization. Through its well-considered architecture for feature information processing and storage mechanisms, OSA demonstrates significant potential for enhancing both system performance and energy efficiency, while effectively addressing the requirements of real-world application scenarios.

### Multi-layer feature fusion architecture

**Fig 7:**
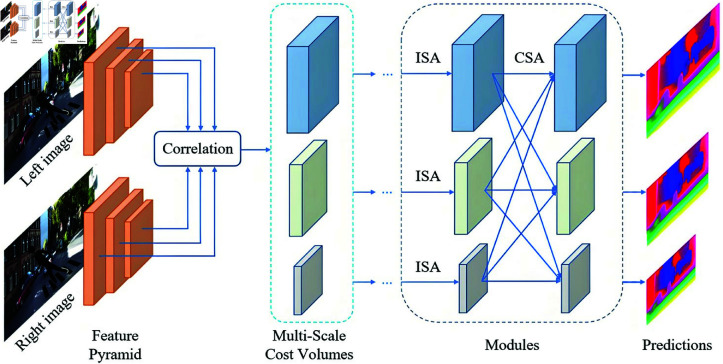
Schematic diagram of multi-layer feature fusion structure.

In complex lighting and weather conditions, pedestrian detection becomes significantly more challenging due to severe feature degradation across multiple dimensions. Conventional pedestrian detection methods, which are typically optimized for normal lighting and weather scenarios, exhibit substantially higher rates of both missed detections and false positives when applied to such challenging environments. To address the limitations of existing detection methods in complex scenarios, this study proposes a novel approach incorporating a multilayer feature fusion architecture, which effectively enhances the detection capability for pedestrians that are otherwise undetectable by traditional methods. A schematic diagram of the multi-layer feature fusion structure is shown in Fig 7.

To enhance pedestrian detection performance under challenging environmental conditions, we propose a novel multilayer feature fusion architecture. This structure meticulously integrates illumination-robust pedestrian information with distinctive body shape characteristics, enabling reliable hypothesis region generation across diverse lighting and weather scenarios. The system serves as a robust complement to conventional pedestrian tracking and detection methods, particularly effective in normal environmental conditions. Furthermore, our approach incorporates an intelligent adaptive adjustment mechanism that dynamically optimizes model parameters and strategies based on real-time analysis of video stream attributes, including image quality metrics and target feature characteristics. To bolster the model’s adaptability, we implement comprehensive data augmentation techniques that significantly expand the diversity of training datasets, thereby enhancing the model’s generalization capabilities across various operational environments. This optimized architecture demonstrates superior detection accuracy while maintaining computational efficiency, making it particularly suitable for real-world surveillance applications where environmental conditions are frequently variable and unpredictable.

## Experimental results and analysis

This experiment is based on the improved YOLOv8 and OFEO network with a Windows environment, Python 3.6.13 as the development language, NVIDIA GeForce RTX 2070 SUPER (8G) as the GPU, and Intel(R) Core(1TM) i5-10500 CPU@3.10GHz as the CPU configuration.

### Experimental parameter settings and implementation details

The S-YOFEO framework incorporates several key enhancements to improve small object detection and tracking performance. In the YOLOv8 architecture, a dedicated small target detection head was integrated, and the input resolution was increased to 1280×1280 pixels to better capture small objects. The model was trained with a learning rate of 0.001, a batch size of 16, and optimized over 300 epochs using a momentum of 0.9 and a weight decay of 0.0005.

For feature extraction in the DeepSORT framework, OSNet replaced the original network, with its feature dimension set to 512. OSNet was pre-trained on the Market-1501 dataset for person re-identification tasks, using a learning rate of 0.0003 and a batch size of 64. The FSA algorithm was implemented with an initial process noise covariance of 0.01 and an initial measurement noise covariance of 0.1. The forgetting factor was dynamically adjusted within the range [0.95, 1.0] based on the motion consistency of tracked objects, enhancing adaptability to varying motion patterns.

In the association stage, EIOU replaced the traditional CIOU as the association metric in DeepSORT, with a matching threshold set to 0.5. Tracks were considered lost after 30 consecutive missed detections, balancing robustness and computational efficiency. OSA was employed as the trajectory feature extractor, with a feature dimension of 256. Its single-pass aggregation mechanism significantly reduced computational overhead while maintaining feature richness. OSA was trained with a learning rate of 0.0001 and a batch size of 32.

Ablation studies were conducted to validate the contribution of each component in the S-YOFEO framework. The results demonstrated that the small target detection head and OSNet integration significantly reduced ID switches and fragmentation, improving tracking continuity. The FSA algorithm and EIOU metric enhanced robustness in complex scenarios, while OSA optimized computational efficiency without sacrificing accuracy. These enhancements led to measurable improvements in both tracking accuracy and speed, validating the effectiveness of the proposed framework.

### Experimental data sets and evaluation indexes

CrowdHuman [27] and DukeMTMC-reID [28] are the training datasets utilized in this paper. MOT16 [29] and MOT17 [30] are the test datasets. The DukeMTMC-reID dataset is used to train pedestrian feature information extraction, the CrowdHuman dataset is used to train target pedestrian detection, and MOT16 and MOT17 are used to assess the S-YOFEO model’s tracking performance. There are a total of 470,000 instances in the training and validation sets of the CrowdHuman dataset, roughly 23 individuals per image with 15,000 sheets in the training set, 5,000 sheets in the test set, and 4,370 sheets in the validation set. The DukeMTMC-reID dataset comprises 2,228 images in the validation set, 702 pedestrians plus 408 obstructing pedestrians in the test set (17,661 photos), and 702 pedestrians in the training set (16,522 images). MOT16 and MOT17 have fourteen video segments apiece.

Five common evaluation metrics for multi-target tracking—Multiple Object Tracking Accuracy (MOTA) [31], Identification F-Score (IDF1) [32], Mostly Tracked (MT) [33], FPS and Identity Switching Rate (IDSW) [34], are chosen in this paper in order to objectively assess the performance of the S-YOFEO model from all angles.The specific meanings are shown in Table 1.

**Table 1:** Five multi-target tracking evaluation indexes.

Indicators	Meaning
MOTA	Tracking accuracy measures the degree of target trajectory retention and the overall performance of the model.
IDF1	Measure the retention of tracker IDs.
MT	The number of GT tracks successfully tracked in the entire video accounts for more than 80% of the total frame count.
FPS	Evaluate the detection speed of the model.
IDSW	Measure how often the tracker switches between different targets.

### Analysis of experimental results of the S-YOFEO model

This paper utilizes the OSNet network for pedestrian tracking training to compare its accuracy and efficiency with other multi-target feature extraction networks, as indicated in Table 2, in order to fairly assess the final tracking impact.

**Table 2:** The performance of YOFGD model on MOT16.

Feature Extraction Network	Weight Size /MB	Average Accuracy /%
OSNet	2.45	83.068
ShuffletNet_V2	2.56	76.171
Resnet50	95.77	74.829
Resnet101	168.01	73.503
CNN	43.89	76.554

**Fig 8:**
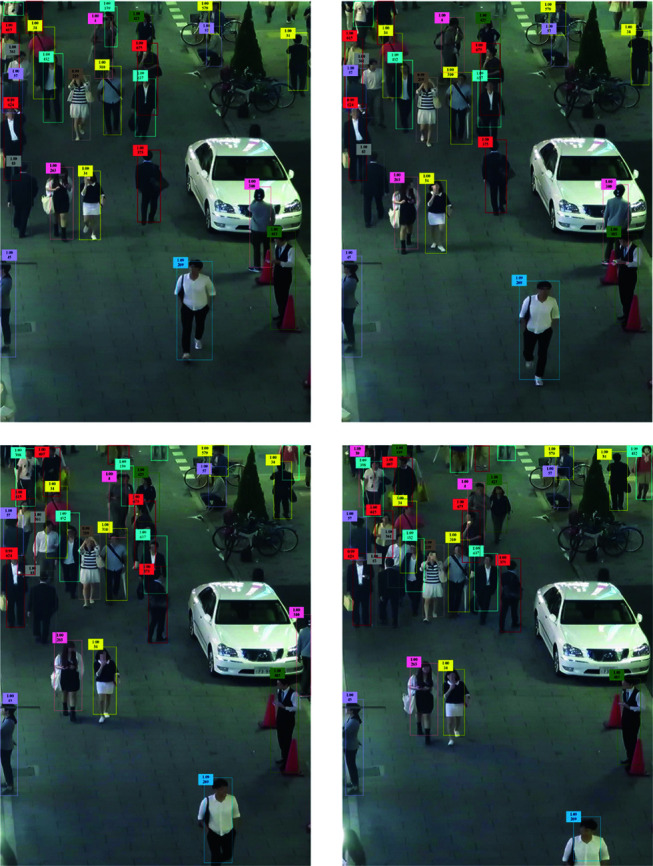
Partial tracking results of MOT17 dataset.

In this paper, some sequences that are taken by drones in MOT17 are selected for visualization, as shown in Fig 8.

According to the results shown in Fig 8, the S-YOFEO model is still able to track pedestrians with partial occlusions and localize them in densely populated regions without misdetection, omission, or ID switching, even in complicated situations.

The model of YOLOv8 paired with Deepsort was chosen for experimental comparison with the S-YOFEO model established in this paper in order to better illustrate the differences between the various tracking methods. It is evident that even in complicated settings, the S-YOFEO model continues to perform well in terms of tracking.

In this study, the MOT16 and MOT17 datasets are used to test the S-YOFEO and the test results are displayed in Table 3 and Table 4.

**Table 3:** The performance of S-YOFEO model on MOT16.

Algorithm	MOTA↑	IDF1↑	MT↑	FPS↑	IDSW↓
S-YOFEO	69.9	71.5	41.6	56.0	713
YOLOv8+					
CNN+Deepsort	61.5	62.3	34.8	57.4	776
JDE-864	62.2	57.0	34.5	24.2	1605
JDE-1088	64.1	55.6	35.2	18.7	1542
FairMOT	68.8	70.3	39.3	25.6	954
Tracktor	54.4	52.5	18.9	5.1	1030
CenterTrackPub*	67.7	57.1	32.9	6.8	947

**Table 4:** The performance of S-YOFEO model on MOT17.

Algorithm	MOTA↑	IDF1↑	MT↑	FPS↑	IDSW↓
S-YOFEO	78.2	76.3	45.6	48.8	622
YOLOv8+					
CNN+Deepsort	67.4	55.9	36.4	56.3	1543
JDE-864	66.5	57.2	32.8	19.5	1901
JDE-1088	67.6	57.5	32.2	18.3	1844
FairMOT	67.7	70.1	37.7	18.8	2189
Tracktor	56.3	52.2	19.6	25.1	2075
CenterTrackPub*	61.6	53.4	26.3	17.9	5327

According to the experiments, S-YOFEO obtains 69.9% MOTA and 713 IDSW on the MOT16 dataset, and 78.2% MOTA and 713 IDSW on the MOT17 dataset. The aforementioned data demonstrate that, while missing the fastest tracking speed, the S-YOFEO model performs better overall and maintains its leading speed. In summary, S-YOFEO has improved somewhat in terms of overall effectiveness and tracking.

## Conclusion

This work proposes a novel multi-target pedestrian tracking technique for complicated environments that is innovative, based on optimized DeepSORT and improved YOLOv8. Aiming the current multi-target tracking algorithms in the CrowdHuman dataset, which features pedestrians crammed into close quarters with mutual occlusion, this paper enhances the feature extraction network, Kalman filter, and IOU, which are sequentially enhanced to significantly improve the robustness and adaptability of the tracking system while maintaining the accuracy and efficiency of the model. First off, by using OSNet rather than the traditional CNN, this paper’s lightweight feature extraction network greatly increases the efficiency and accuracy of target pedestrian recognition, and this change not only reduces the operational burden of the model but also provides the possibility of real-time tracking applications. The tracker’s accuracy is then increased by applying the FSA Kalman filter, a new adaptive oblivious Kalman filter technique; the tracking errors due to target occlusion, fast movement, and other factors are effectively reduced. The match between the detection and prediction frames is then measured using an EIOU correlation matching metric to enhance the tracking accuracy even more. Lastly, to enhance the tracker’s capacity to gather global data and derive the desired trajectory, a generalized trajectory feature extraction network OSA is employed. The S-YOFEO model presented in this paper consistently improves tracking speed and accuracy in experiments.

In complex traffic environments, interactions and occlusions between multiple targets may lead to data correlation problems and increase the difficulty of tracking. Although S-YOFEO has achieved high accuracy and speed, it may still face challenges when dealing with highly dense and fast-moving targets. In particular, whether the S-YOFEO model can maintain the same excellent tracking results under different brightness, weather conditions, and other complex and changing external environments is one of the important directions of current research. Future work will be devoted to exploring the adaptability of the S-YOFEO model in different scenarios. By researching more efficient data association algorithms and tracking strategies, to improve the system’s performance when dealing with highly dense and fast-moving targets. Through algorithm optimization and hardware design, the demand for computational resources of S-YOFEO is reduced to achieve efficient operation even in resource-constrained environments. Promote the development of multi-target pedestrian tracking technology to a higher level through continuous technology iteration and optimization.

## Supporting information

S1 MOT Minimal test data set (ZIP)
