# Supplementary figures and images for: Pedestrian tracking method based on S-YOFEO framework in complex scene
Source: PLoS One. 2025 Jun 4;20(6):e0322919. doi: 10.1371/journal.pone.0322919 (PMC12136331; doi:10.1371/journal.pone.0322919)

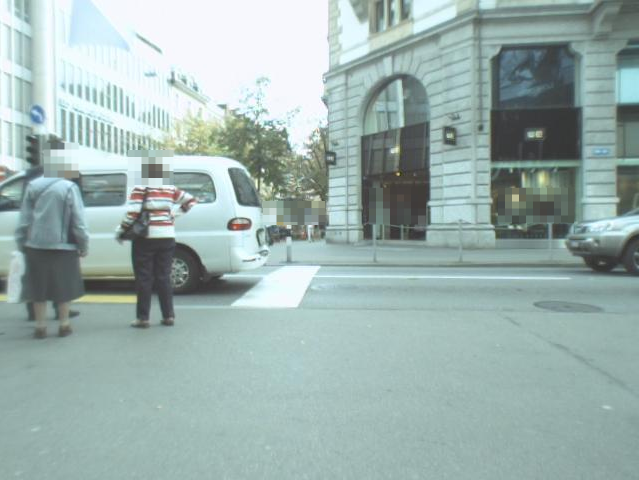

Supplement: S1 MOT — (ZIP) [file pone.0322919.s001.zip › MOT-Revised/000001.png]

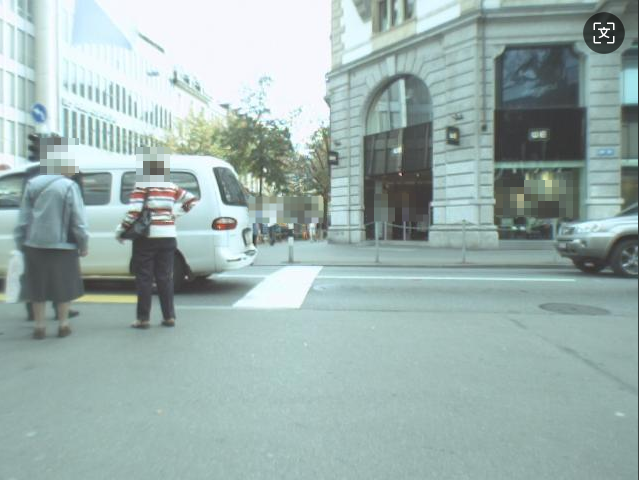

Supplement: S1 MOT — (ZIP) [file pone.0322919.s001.zip › MOT-Revised/000002.png]
